# Supplementary material for: Prevalence and pre-disposing factors of Helicobacter pylori among patients with gastro-intestinal symptoms attending Mulago Hospital, Kampala, Uganda
Source: PLoS One. 2026 Jul 15;21(7):e0342871. doi: 10.1371/journal.pone.0342871 (PMC13372166; doi:10.1371/journal.pone.0342871)
Supplement: S1 File — (DOCX) [file pone.0342871.s001.docx]

## Additional Information

## INFORMED CONSENT FORM FOR RESEARCH STUDY

SCHOOL OF PUBLIC HEALTH MAKERERE UNIVERSITY

**STUDY TITLE:** PREVALENCE AND PRE-DISPOSING FACTORS OF HELICOBACTER PYLORI AMONG PATIENTS WITH GASTRO-INTESTINAL SYMPTOMS ATTENDING MULAGO HOSPITAL, KAMPALA UGANDA

**PRINCIPAL INVESTIGATOR:** TWIKIRIZE RACHEAL

**INTRODUCTION:**

You are invited to participate in a research study on the prevalence of Helicobacter pylori (H. pylori) infection and its predisposing factors among patients with gastrointestinal symptoms. Before you decide whether to participate, you must understand the purpose, procedures, risks, benefits, and confidentiality of this study. Please take your time to read this form carefully and feel free to ask any questions you may have.

1. **Study Purpose:**

The purpose of this study is to determine the prevalence of H. pylori infection and identify potential predisposing factors associated with gastrointestinal ulcers at Mulago Hospital. The findings from this research may contribute to a better understanding of H. pylori-related diseases and facilitate the development of more effective prevention and treatment strategies.

1. **Procedures:**

Participation in this study will involve answering a questionnaire about your medical history, lifestyle habits, and other relevant information. A non-invasive test, such as a stool sample, will be used to detect the presence of H. pylori infection.

All information collected will be kept confidential and used solely for research purposes.

1. **Potential Risks:**

The risks associated with participating in this study are minimal. Some individuals may experience mild discomfort or inconvenience during the data collection process since some individuals might have difficulties in producing a sample and it might take time and effort, in addition to feeling unfamiliar and awkward. However, the research team will take necessary measures to minimize any potential risks.

1. **Benefits:**

While there are no direct benefits to you as a participant, your involvement in this study will contribute to advancing medical knowledge regarding H. pylori infection and gastrointestinal ulcers. The results obtained from this research may potentially benefit individuals in the future by improving diagnostic and treatment methods.

- 1. **Confidentiality:**

Your privacy is of utmost importance to us. All data collected during this study will be treated with strict confidentiality. Personal identifiers will be removed, and your data will be stored securely with access restricted to the research team only. The results of this study will be presented in aggregate form, ensuring individual anonymity.

- 1. **Voluntary Participation:**

Participation in this study is entirely voluntary. You have the right to decline to participate, and your decision will not affect your relationship with the research team. If you decide to participate, you may withdraw your consent and discontinue your involvement at any time without penalty or loss of benefits.

- 1. **Contact Information:**

If you have any questions or concerns regarding this study, please feel free to contact the Principal Investigator, Twikirize Racheal, on (…). For questions about your rights as a study participant, contact the MakSPH Higher Degrees Research &Ethics Committee Chairperson, Dr. Joseph Kagaayi on (…).

By signing below, you acknowledge that you have read and understood the information provided in this consent form, and you voluntarily agree to participate in the research study.

Participant's Signature: Date:

Name of Participant:

Interviewers Signature: Date:

## Questionnaire

Dear respondents, you are requested to answer the questions on this interview guide as required or as will be asked of you. Your honesty and truthfulness will render this study a success. I will use a code as your identification number other than your name.

Please tick the appropriate response

| **Participant Information** |  |
| --- | --- |
| 1. Participant ID | [To be filled] |
| 2. Name |  |
| 3. Age |  |
| 4. Address |  |
| 5. Where you Referred? If yes, Where from? |  |
|  |  |
| **Section 1: Demographics** |  |
| 1. Gender | [ ] Male |
|  | [ ] Female |
|  |  |
| 2. Marital Status | [ ] Single |
|  | [ ] Married |
|  | [ ] Separated |
|  |  |
| 3. Educational Level | [ ] Primary |
|  | [ ] Secondary |
|  | [ ] Tertiary |
|  | [ ] Other: |
|  |  |
| 4. Employment status | [ ] Employed |
|  | [ ] Self-Employed |
|  | [ ] Unemployed |
|  |  |
| 5. What is your monthly household income after taxes | [ ] Less than Ugx 500,000/= |
|  | [ ] Ugx 500,000 to 1,000,000/= |
|  | [ ] Ugx 1,000,000 to 1,500,000/= |
|  | [ ] Above Ugx 1,500,000/= |
|  |  |
| 6. How many people are financially dependent on this income | [ ] None |
|  | [ ] 1 to 5 |
|  | [ ] 5 to 10 |
|  | [ ] Above 10 |
|  |  |
| **Section 2: Lifestyle and Dietary Habits** |  |
| 1. Do you smoke tobacco products? | [ ] Yes |
|  | [ ] No |
| If yes, indicate average number per day and duration: |  |
|  |  |
| 2. Do you consume alcohol? | [ ] Yes |
|  | [ ] No |
| If yes, indicate frequency and average amount per week: |  |
|  |  |
|  |  |
| 3. How often do you consume spicy or acidic foods? | [ ] Daily |
|  | [ ] Weekly |
|  | [ ] Monthly |
|  | [ ] Rarely |
|  | [ ] Never |
|  |  |
| 4. Do you wash your hands before eating food or fruits? | [ ] Always |
|  | [ ] Often |
|  | [ ] Never |
|  |  |
| 5. Where do you always eat from? | [ ] Home |
|  | [ ] Restaurant |
|  | [ ] Road-side |
|  | [ ] Others |
| 6. Where do you collect your water from? | [ ] Tap |
|  | [ ] Tank |
|  | [ ] Stream |
|  | [ ] Borehole |
|  |  |
| **Section 3: Medical History** |  |
| 1. Ever diagnosed with a gastrointestinal ulcer? | [ ] Yes |
|  | [ ] No |
| If yes, provide details: |  |
|  |  |
| 2. Ever tested for H. pylori infection? | [ ] Yes |
|  | [ ] No |
| If yes, provide details: |  |
|  |  |
| 3. Ever received treatment for H. pylori infection? | [ ] Yes |
|  | [ ] No |
| If yes, provide details: |  |
|  |  |
|  |  |
| **Section 4: General Health and Medications** |  |
| 1. Are you currently taking any medications? | [ ] Yes |
|  | [ ] No |
| If yes, list medications and dosage: |  |
|  |  |
| 2. Used NSAIDs (e.g., aspirin, ibuprofen) in the last 1 month? | [ ] Yes |
|  | [ ] No |
|  |  |
| 3. Did you have any of the following symptoms? | [ ] Abdominal Pain |
|  | [ ] Bloating and Fullness |
|  | [ ] Nausea and Vomiting |
|  | [ ] Heartburn and Acid Reflux |
|  | [ ] Fatigue |
|  |  |
| **Section 5: Family History** |  |
| 1. Immediate family members diagnosed with H. pylori or gastrointestinal ulcers? | [ ] Yes |
|  | [ ] No |
| If yes, specify the relationship and condition: |  |
|  |  |
| **Section 6: Stool Results** | [ ] Positive |
|  | [ ] Negative |
|  |  |
| **Section 7: Additional Comments** |  |
| Additional comments or information: |  |
|  |  |
|  |  |
|  |  |
